# Percutaneous radiofrequency thermocoagulation and microvascular decompression for treating glossopharyngeal neuralgia: a retrospective clinical study

**DOI:** 10.1186/s12883-023-03415-z

**Published:** 2023-10-23

**Authors:** Zeyu Wu, Yongming Zhao, Fan Wu, Yiyue Fan, Ying Yang

**Affiliations:** 1 Department of Pain,The Affiliated Nanchong Central Hospital of North Sichuan Medical College, 97, South Renmin Road, Shunqing District, Nanchong, Sichuan China; 2 Department of Pharmacy, Sichuan Nanchong Mental Health center, The Second People’s Hospital of Nanchong, Nanchong, Sichuan China

**Keywords:** Glossopharyngeal neuralgia, Percutaneous radiofrequency thermocoagulation, Microvascular decompression, Curative effect, Prognosis

## Abstract

**Objectives:**

This study aimed to investigate the differences in the effectiveness of percutaneous radiofrequency thermocoagulation (PRT) and microvascular decompression (MVD) in treating glossopharyngeal neuralgia (GPN).

**Methods:**

Medical records of patients were reviewed to investigate their baseline characteristics and immediate postoperative prognosis. Long-term outcomes of these patients were obtained through telephone interviews. Visual analog scale (VAS) and Pittsburgh sleep quality index (PSQI) scores at 1 day and 1, 4, 12, 24, and 48 weeks after surgery were compared between the MVD and PRT groups, in addition to complete pain relief rate, effective rate, adverse reactions, length of hospital stay, and economic indicators.

**Results:**

The VAS and PSQI scores of the two groups at 1 day and 1, 4, 12, 24, and 48 weeks after surgery were significantly lower (*P* < 0.05) than those before surgery. At 48 weeks, the complete remission rate was significantly higher (*P* < 0.05) in the MVD group than in PRT group. No significant difference in adverse reactions was observed between the two groups. The length of hospital stay, operative time, and cost were significantly higher (*P* < 0.05) in the MVD group than in the PRT group.

**Conclusions:**

Both PRT and MVD can significantly reduce patients’ degree of pain and improve their sleep quality. In the medium term, MVD is better than PRT in terms of the complete curative effect. In young patients with GPN, MVD is more often recommended than PRT; however, MVD is costlier than PRT.

## Introduction

The glossopharyngeal nerve is the ninth pair of cranial nerves within the intracranial region. Glossopharyngeal neuralgia (GPN) is characterized by transient episodes of pain in the distribution areas of the glossopharyngeal nerve, which include the mandibular angle, ear, tonsillar fossa, posterior pharyngeal area, and base of the tongue. This pain typically manifests as stabbing sensations and is often triggered by activities such as coughing, talking, swallowing, and yawning [1]. The prevalence of GPN is estimated at 0.8 per 100 000 individuals per year and tends to increase with age, most commonly occurring in adults aged > 50 years. While trigeminal nerve compression typically occurs on the right side, the glossopharyngeal nerve is more commonly affected on the left side [2, 3]. GPN is likely caused by compression of the glossopharyngeal nerve by vessels in the inlet area of the brainstem root [4]. Other potential causes of GPN involve trauma, tumors (such as those at the skull base, pontocerebellum, brainstem, pharynx, tongue, and tonsil as well as metastatic head and neck tumors), infections (including tonsillitis, pharyngitis, arachnoiditis, parapharyngeal abscess, and tuberculosis), and surgery. The pain experienced during GPN may be accompanied by additional symptoms, such as numbness [5]. The pain during a GPN attack is severe, and long-term pain can induce lesions in other organs, thereby causing significant physical and psychological harm to the patient, severely impacting their quality of life, and posing potential risks to society. GPN can be treated using various approaches, including conservative and surgical treatments. In conservative treatment, antiepileptic drugs such as carbamazepine are used. These drugs stabilize the nerve cell membrane and reduce its permeability to Na + and Ca2+, thereby decreasing cell excitability and prolonging the refractory period [6]. A nerve block is performed via the lateral cervical approach [7]. Meanwhile, surgical treatment approaches for GPN include pulsed radiofrequency [8], percutaneous radiofrequency thermocoagulation(PRT) [9], microvascular decompression(MVD) [10], and stereotactic body radiation therapy [11]. These treatments aim to relieve pain and improve patients’ sleep and quality of life. Previous studies [7–11] have demonstrated the effectiveness of these treatments for GPN; however, their advantages and disadvantages have not been compared. Therefore, this study aimed to compare the mid-term clinical efficacy of PRT and MVD in treating GPN to provide a reference for patients to choose a better treatment plan.

## Materials and methods

### Patients

In this retrospective study, clinical data of patients with a clear diagnosis of GPN admitted to the Pain Management and Research Center of the Second Clinical College of Chuanbei Medical College (Nanchong Central Hospital) from January 2015 to January 2021 were collected. The patients were divided into the PRT and MVD groups according to the different treatment methods used. All participants provided written informed consent before being enrolled in the study. The study was approved by the Ethics Committee of Nanchong Central Hospital [Review (001) No. 2021] and was conducted according to the Declaration of Helsinki.

### Inclusion and exclusion criteria

The inclusion criteria were as follows: (1) patients with clinically confirmed GPN; (2) those with a pain duration of > 6 months; (3) those receiving continuous treatment with standard analgesic drugs and nerve blocks without improvement in pain and a visual analog scale (VAS) score of > 5; (4) those receiving PRT or MVD during hospitalization (patients who underwent PRT exhibited the following clinical features: (1) severe systemic diseases with poor control; (2) intolerance to general anesthesia required for craniotomy; and (3) inability to comprehend and prepare for the potential efficacy and complications associated with craniotomy. Conversely, patients who underwent MVD exhibited the following characteristics: (1) magnetic resonance functional imaging showed compression of the glossopharyngeal nerve by offending vessels; (2) ability to comprehend the surgical methods and associated risks; and (3) poor response to PRT). The exclusion criteria were as follows: (1) patients treated with medications or nerve block alone; (2) those with inadequate case information to complete follow-up; and (3) those with other postoperative comorbidities who could not correctly describe their current status and complete the relevant score.

### Treatment modality

**Fig. 1:**
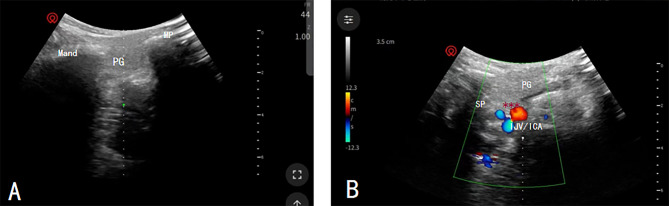
**A** Bone localization marker sonogram; **B** Color Doppler sonogram under the puncture plane. Notes: ***indicates RFT needle. Abbreviations: Mand, mandibular angle; MP, Mastoid process; PG, Parotid gland; SP, styloid Process; IJV/ICA, Internal jugular vein/internal carotid artery

In the PRT group, the patient was positioned supine with established intravenous access. After cardiac monitoring, the head was slightly tilted toward the healthy side. A high-frequency linear array ultrasound probe (Watson Compass NaviX) was placed midway between the mastoid and the angle of the mandible. The probe was routinely disinfected, covered with a sterile film, and oriented in an out-of-plane approach, with the ultrasound plane close to the horizontal section. This allowed for observation of the parotid gland, with the probe adjusted downward as needed. The parotid gland was observed until it disappeared from view, revealing the deeper portion of the gland, which extended to the styloid process of the temporal bone. The tip of the styloid process of the temporal bone served as the initial puncture target, and local infiltration anesthesia was administered. Under ultrasound guidance, puncture was performed using a 22-G radiofrequency needle, with the needle depth marked when the tip reached the styloid process. If the offending vessels could not be reached near the styloid process, the approach was directed toward the internal carotid artery. The needle was slowly retracted, allowing the tip to bypass toward the internal carotid artery, and then it was punctured at a depth of approximately 0.3–0.7 cm. Back injection was performed to verify the absence of blood and cerebrospinal fluid on the radiofrequency electrode (Beiqi Company model: R-2000BD1) used for testing. In the first sensory test, the parameters were as follows: pulse width, 0.1 ms; frequency, 50 Hz; and voltage, 0.5 volts. The needle tip was adjusted until the sensory stimulation could induce sensation in the patient’s glossopharyngeal nerve area. If the needle tip position was deemed satisfactory, a motor test was performed. The parameters for the motor test were as follows: pulse width, 3 ms; frequency, 2 Hz; and voltage, 0.5 volts. This stimulation was capable of inducing pharyngeal muscle contraction. The needle was slowly adjusted to achieve maximum sensation, and the needle tip position was deemed satisfactory. After confirming that there was no blood and cerebrospinal fluid via back injection, treatment was administered using intravenous propofol (1.5–2.0 mg/kg). When the patient became unconscious, standard continuous radiofrequency was applied at temperatures of 70 °C, 75 °C, and 80 °C, each for a duration of 180 s. Following the radiofrequency treatment, the patient was awakened, and a 2 mL anti-inflammatory analgesic solution (compound betamethasone [2 mg], 2% lidocaine [1 mL], and 0.9% saline [1 mL]) was injected into the puncture needle. After the needle was removed, pressure was applied to the puncture site to control bleeding and then covered with a sterile dressing (Fig. 1).

In the MVD group, the retrosigmoid suboccipital approach was employed. The patient was positioned in the lateral decubitus position, with the mastoid root aligning with the highest point of the surgical area. The head frame was securely fixed, and a 5-cm arc incision was made along the posterior inferior occipital sigmoid sinus. After disinfection and placement of a sterile towel, an incision was made through the skin and muscles, extending straight to the occipital squamous area. A mastoid spreader was utilized to open the area, and access to the suboccipital sigmoid sinus was achieved using a high-speed grinding drill. This resulted in the formation of a bone window measuring approximately 2.0 × 2.5 cm, with the upper edge positioned 0.5–1.0 cm from the transverse sinus and the anterior outer edge situated in close proximity to the posterior edge of the sigmoid sinus. The dura was incised in an arc, followed by microdissection of the arachnoid around the posterior group of cerebral nerves. The pontocerebellar angle cistern was then opened, allowing sufficient release of cerebrospinal fluid to expose the brainstem and access the posterior group of cerebral nerves. The gap above the facial auditory nerve was explored, and the responsible vessels were carefully separated. A Teflon spacer was inserted between these vessels and the root of the glossopharyngeal nerve as it exited the brainstem. This area was probed to ensure there was no remaining vascular compression. After confirming the absence of active bleeding in the operative area, the wound was thoroughly flushed with saline to satisfaction. The dura was tightly sutured, and the procedure was concluded with layer-by-layer suture dressing.

### Efficacy assessment

1. Pain level: VAS was used to record the level of pain before surgery and at 1 day and 1, 4, 12, 24, and 48 weeks after surgery (0 indicates no pain and 10 indicates intolerable pain; the higher the score, the more severe the pain).
2. Sleep quality: The Pittsburgh sleep quality index (PSQI) was used to record sleep quality. Scores (ranging 0–21) were assessed before surgery and at 1 day and 1, 4, 12, 24, and 48 weeks after surgery (the lower the score, the better the sleep quality).
3. Pain relief: This was assessed based on two criteria: the complete pain relief rate (no pain and no analgesic use) and the effective rate of pain relief (pain reduction of > 50% from the baseline).
4. Adverse reactions: The incidence of postoperative adverse reactions was compared between the two groups.
5. Others: Operative time, length of hospital stay, and total cost of hospitalization were compared between the two groups.

### Statistical analysis

Statistical Package for the Social Sciences (version 25.0) was used for all data analyses, and GraphPad Prism 8 was used for creating graphs. Measurement data conforming to a normal distribution are presented as means ± standard deviations $(\bar x\, \pm \,s)$. Independent sample t-test was used to compare the groups. Measurement data with a skewed distribution are expressed as medians and interquartile ranges [M (P_25_–P_75_)]. Comparison between groups was performed using Mann–Whitney U test. Repeated-measures data were analyzed using repeated-measures analysis of variance. The enumeration data are expressed as [*n* (%)], and the groups were compared using chi-square test or Fisher’s exact test because the sample size was < 40. The test level α was 0.05; *p*-values of < 0.05 were used to indicate statistical significance.

## Results

### Patients’ basic clinical data

**Fig. 2:**
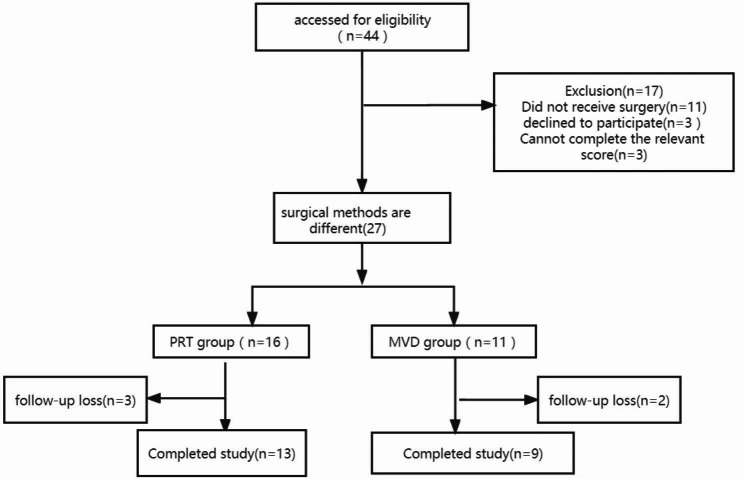
Study flowchart. Abbreviations: GPN, glossopharyngeal neuralgia; PRT, percutaneous radiofrequency thermocoagulation; MVD, microvascular decompression

From January 2015 to January 2021, 44 patients with a clear diagnosis of GPN were assessed at the Pain Management and Research Center, Nanchong Central Hospital. A total of 22 patients, including 11 who did not undergo surgery, 5 who were lost to follow-up, 3 who declined to participate, and 3 who could not correctly describe their postoperative status and complete the relevant score, were excluded. Finally, 22 patients met the inclusion criteria. Based on the choice of surgical methods, the patients were divided into the PRT (n = 13) and MVD (n = 9) groups. The flowchart detailing the follow-up procedure is displayed in Fig. 2.

The included 22 patients were aged 41–75 years, with an average age of 62.68 ± 9.12 years (64 ± 8.24 years in the PRT group and 60.78 ± 10.49 years in the MVD group). The median duration of disease onset was 26.5 (range, 17.25–40) months (24 [range, 13–36] months in the PRT group and 26 [range, 18–40] months in the MVD group). Of the 22 patients, 13 were men and 9 were women (8 men and 5 women in the PRT group and 5 men and 4 women in the MVD group) and 7 were left-sided and 15 were right-sided (4 left-sided and 9 right-sided patients in the PRT group and 3 left-sided and 6 right-sided patients in the MVD group). The preoperative VAS score was 7.23 ± 1.19 (7.08 ± 1.26 in the PRT group and 7.44 ± 1.13 in the MVD group). The preoperative PSQI score was 15.14 ± 1.36 (14.85 ± 1.07 in the PRT group and 15.56 ± 1.67 in the MVD group). No significant difference in the patients’ basic clinical data was observed between the two groups (*P >* 0.05; Tables 1 and 2).

**Table 1:** The patients basic clinical data [n, $(\bar x\, \pm \,s)$, M (P_25_-P_75_)]

	PRT group(n = 13)	MVD group (n = 9)	*P* Value
Gender(male/female)	8/5	5/4	0.779
Age(years)	64.00 ± 8.24	60.78 ± 10.49	0.429
Position(Left / right)	4/9	3/6	0.899
Duration of GPN(months)	24(13–36)	26(18–40)	0.593
VAS score pre-operation	7.08 ± 1.26	7.44 ± 1.13	0.491
PSQI score pre-operation	14.85 ± 1.07	15.56 ± 1.67	0.236

**Abbreviations:** GPN, glossopharyngeal neuralgia; PRT, percutaneous radiofrequency thermocoagulation; MVD, microvascular decompression, PSQI, Pittsburgh sleep quality index, VAS, visual analogue scale

**Table 2:** P atient characteristics and the source of the pain

method	Age(y)	Gender	Position	Sources of pain	Duration of GPN(m)	VAS score pre-operation	length of stay(d)	Total cost of hospitalization(¥)	Operative time (min)
PRT	54	M	L	Vascular compression	22	6	7	8254	40
PRT	75	F	R	History of tonsillar and pharyngeal inflammation	8	8	5	7968	40
PRT	75	M	R	Vascular compression	27	9	6	9910	50
PRT	53	M	R	Vascular compression	12	8	10	12,135	60
PRT	67	F	R	After CPA tumor resection	15	7	5	10,181	55
PRT	70	M	L	Vascular compression	18	8	7	8871	65
PRT	64	M	R	Vascular compression	12	7	8	9935	60
PRT	66	M	L	Vascular compression	30	5	6	8125	50
PRT	49	F	R	Vascular compression	36	6	10	15,177	45
PRT	70	F	R	disseminated sclerosis	60	7	6	8325	60
PRT	66	M	R	Vascular compression	40	8	7	8915	65
PRT	58	M	L	Vascular compression	35	8	8	9025	60
PRT	65	F	R	No obvious lesions were found	46	5	7	7835	50
MVD	70	M	R	Responsible blood vessels: PICA	10	9	9	37,128	180
MVD	70	M	L	PICA	50	9	11	39,127	200
MVD	61	F	R	PICA	48	8	13	40,125	210
MVD	41	M	R	PICA combined with the vertebral artery	36	8	20	55,310	220
MVD	53	F	L	PICA	24	7	15	48,325	240
MVD	70	F	R	PICA	40	6	11	41,258	270
MVD	50	M	R	PICA	20	7	9	35,124	300
MVD	67	F	L	PICA combined with the vertebral artery	18	6	14	46,120	210
MVD	65	M	R	PICA	26	7	12	44,100	240

Abbreviations: PRT, percutaneous radiofrequency thermocoagulation; MVD, microvascular decompression GPN, glossopharyngeal neuralgia; PICA, Posterior inferior cerebellar artery

### VAS score before and after surgery

**Fig. 3:**
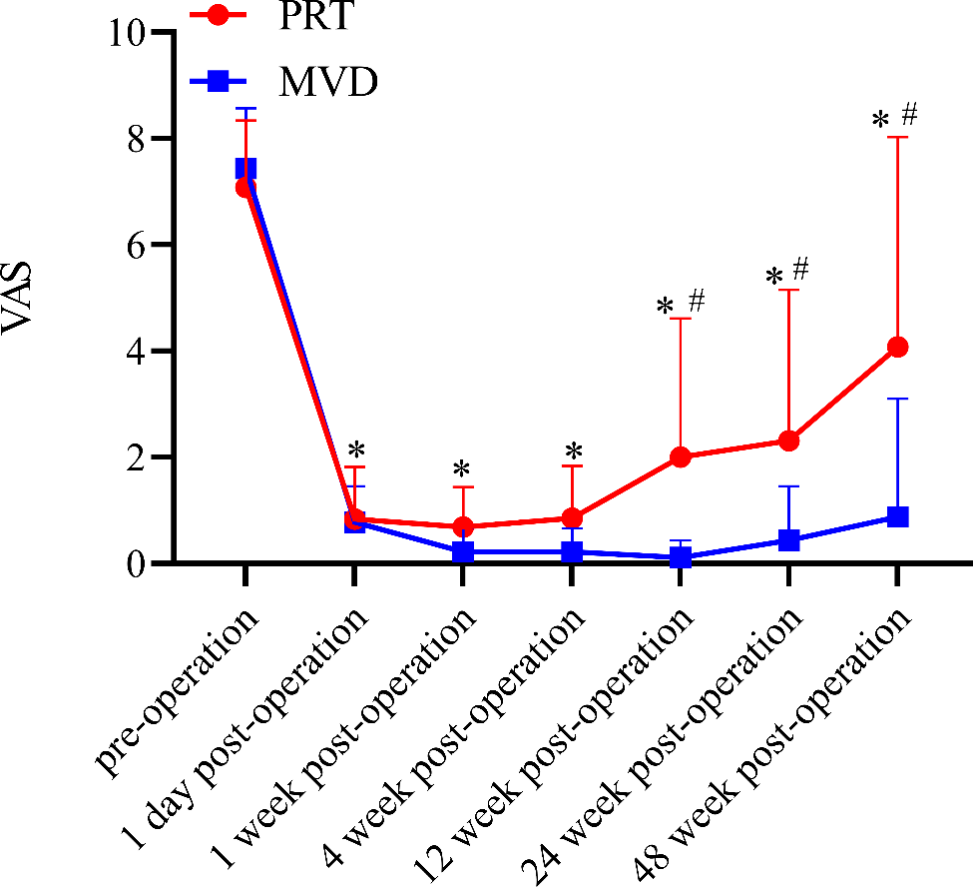
Comparison of VAS score pre- and post operation $(\bar x\, \pm \,s)$. Notes: *P<0.05 indicates post operation VS pre-operation. #*P*<0.05 indicates PRT group vs. MVD group. Abbreviations: PRT, percutaneous radiofrequency thermocoagulation; MVD, microvascular decompression; VAS, visual analogue scale

The VAS scores in the two groups at various time points after surgery were significantly lower (*P* < 0.05) than those before surgery; the VAS scores of the MVD group were significantly lower (*P* < 0.05) than those of the PRT group at 12, 24, and 48 weeks after surgery (Fig. 3).

### PSQI scores before and after surgery

**Fig. 4:**
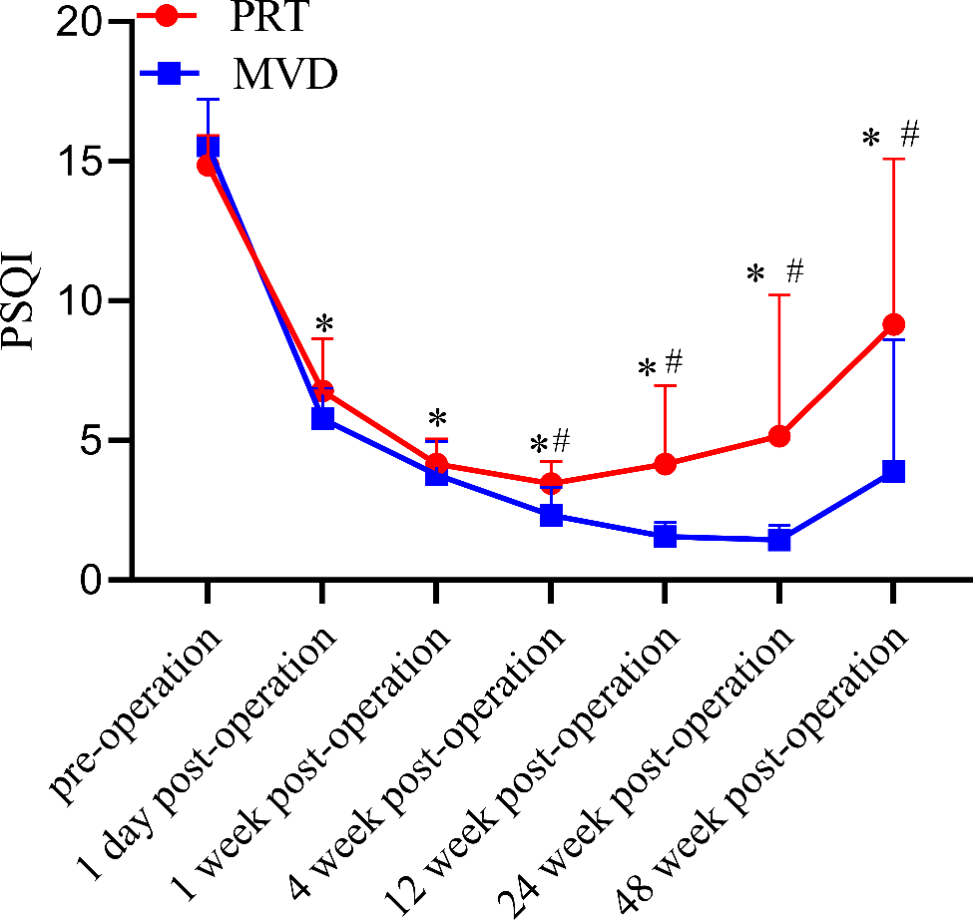
Comparison of PSQI score pre- and post operation $(\bar x\, \pm \,s)$. Notes: *P<0.05 indicates post operation VS pre-operation. #*P*<0.05 indicates PRT group vs. MVD group. Abbreviations: PRT, percutaneous radiofrequency thermocoagulation; MVD, microvascular decompression; PSQI, Pittsburgh sleep quality index

The PSQI scores in the two groups at various time points after surgery were significantly lower (*P* < 0.05) than those before surgery; the PSQI scores of the MVD group were significantly lower (*P* < 0.05) than those of the PRT group at 4, 12, 24, and 48 weeks after surgery (Fig. 4).

### Postoperative complete pain relief rate

**Fig. 5:**
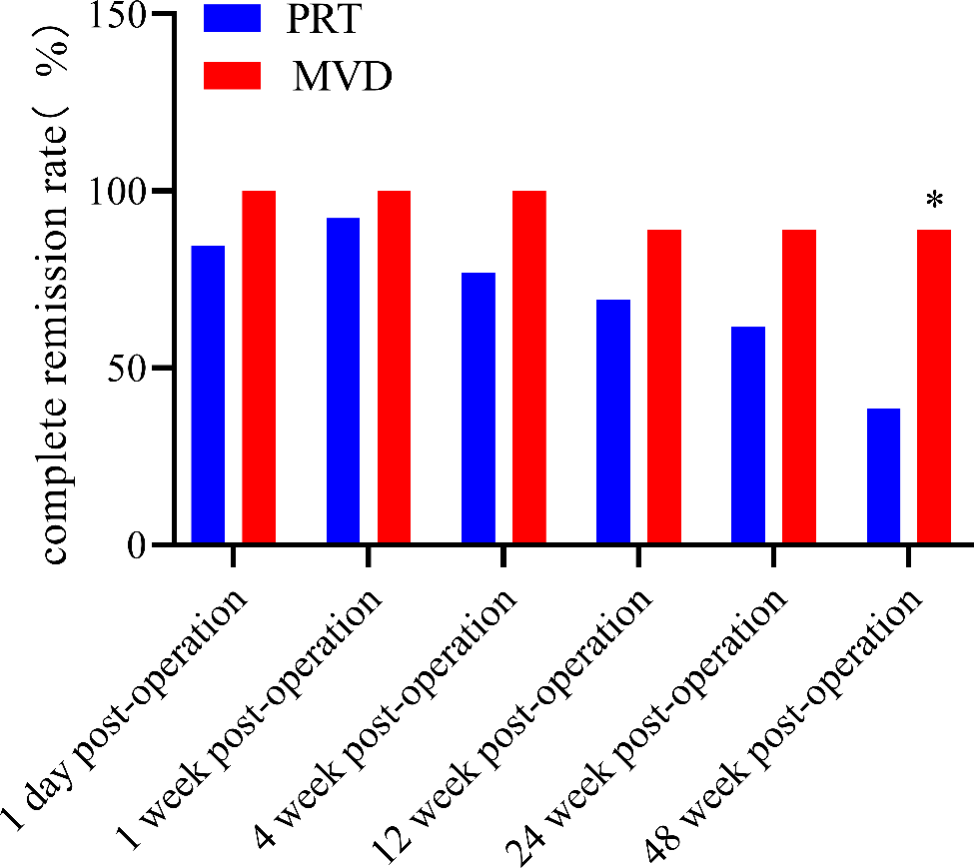
Comparison of 4 Postoperative complete relief rate of pain (%). *P<0.05 indicates PRT group vs. MVD group. Abbreviations: PRT, percutaneous radiofrequency thermocoagulation ; MVD, microvascular decompression

Within 48 postoperative weeks, the complete pain relief rates were 38.5–84.6% and 88.9–100% in the PRT and MVD groups, respectively. At 48 weeks, the complete pain relief rate was significantly higher (*P* < 0.05) in the MVD group than in the PRT group (Fig. 5).

### Postoperative effective rate of pain

**Fig. 6:**
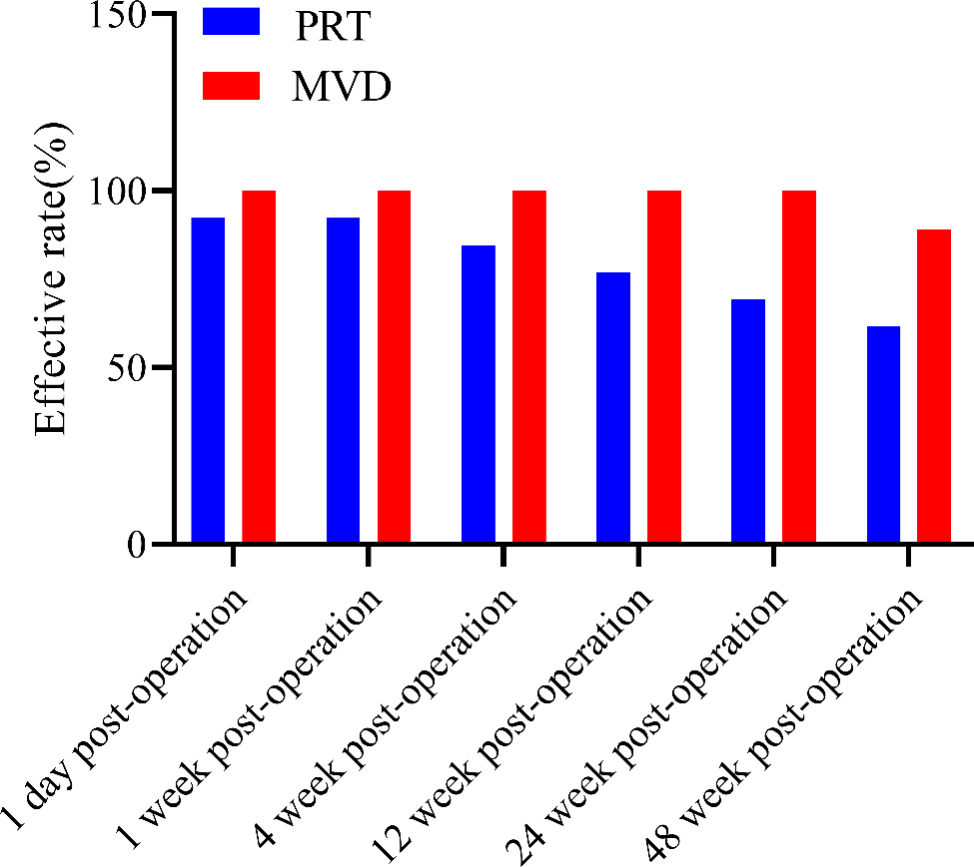
Comparison of Postoperative effective rate of pain (%). Abbreviations: PRT, percutaneous radiofrequency thermocoagulation; MVD, microvascular decompression

Within 48 postoperative weeks, the effective rates of pain were 61.5–92.3% and 88.9–100% in the PRT and MVD groups, respectively, with no significant difference (*P* > 0.05) observed between the groups (Fig. 6).

### Postoperative complications

**Fig. 7:**
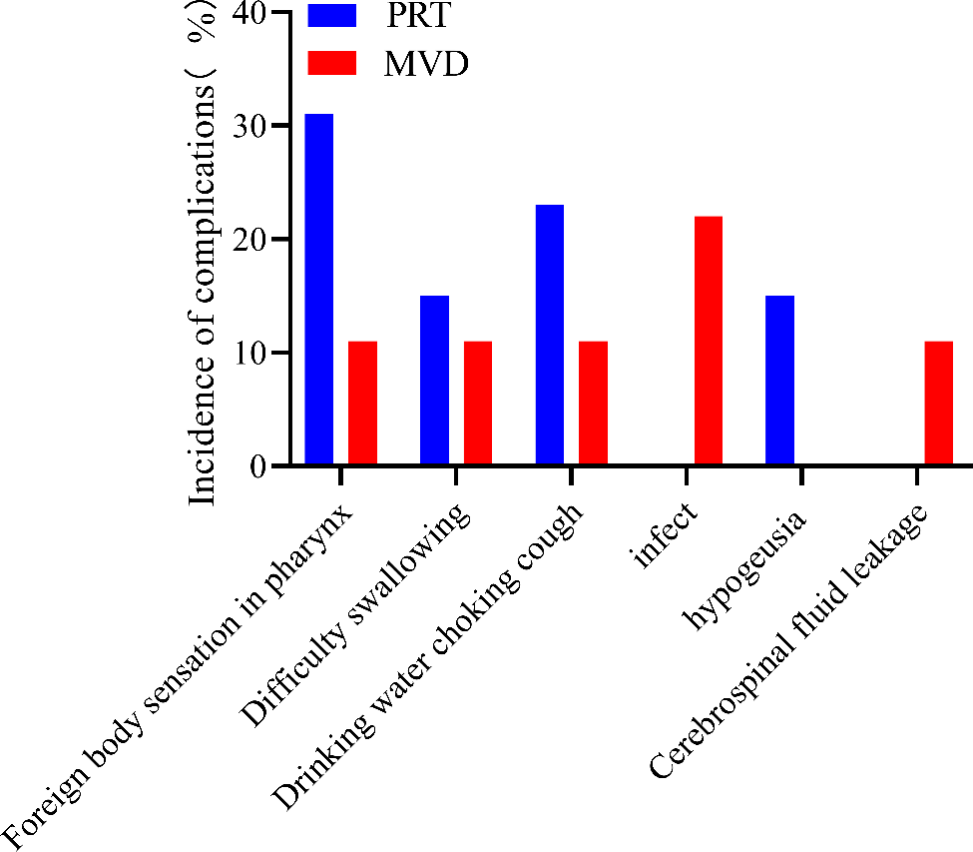
Comparison of Postoperative complications (%). Abbreviations: PRT, percutaneous radiofrequency thermocoagulation; MVD, microvascular decompression

The most common complication after PRT was foreign body sensation in the pharynx (n = 4 patients), whereas that after MVD was infection (n = 2 patients). No significant difference (*P* > 0.05) in the patients’ postoperative complications was observed between the two groups (Fig. 7; Table 3).

**Table 3:** Comparison of Postoperative complications [n(%)]

group	*n*	Foreign body sensation in pharynx	Difficulty swallowing	Drinking water choking cough	infect	hypogeusia	Cerebrospinal fluid leakage
PRT	13	4(30.8)	2(15.4)	3(23.1)	0(0)	2(15.4)	0(0)
order of severity		prolonged the time of hospitalization	prolonged the time of hospitalization	prolonged the time of hospitalization		There was no significant effect on the course of disease	
duration		0-3month	0-1month	0-1month		0-1month	
MVD	9	1(11.1)	1(11.1)	1(11.1)	2(22.2)	0(0)	1(11.1)
order of severity		There was no significant effect on the course of disease	0.00	0.00	prolonged the time of hospitalization		prolonged the time of hospitalization
duration		7 day	3 day	1 day	10 day		7 day

Abbreviations: PRT, percutaneous radiofrequency thermocoagulation; MVD, microvascular decompression

### Operative time, length of hospital stay, and total cost of hospitalization

The MVD group had significantly longer (*P* > 0.05) operative time and length of hospital stay and significantly higher (*P* > 0.05) total hospitalization cost than the PRT group (Table 4).

**Table 4:** Comparison of operative time, length of stay and Total cost of hospitalization [$\bar x\, \pm \,s$, M (P_25_-P_75_)]

group	*n*	Operative time(minute)	length of stay(day)	Total cost of hospitalization(¥)
PRT	13	53.85 ± 8.70	7.08 ± 1.60	8915(8189–10,015)
MVD	9	230.00 ± 37.09	12.67 ± 3.43	41,258(38,128–47,223)
*P* Value		<0.001	<0.001	<0.001

**Abbreviations:** PRT, percutaneous radiofrequency thermocoagulation; MVD, microvascular decompression

## Discussion

GPN is a rare neuropathic pain disorder. The symptoms of GPN were first described by T. Weisenberg in 1910 as recurrent, paroxysmal, sharp, stabbing pain in the tonsils, throat, base of the tongue, and ear canal. This disorder was first named “GPN” by W. Harris et al. in 1921. In 1927, W. Dandy et al. performed the first successful intracranial resection for GPN [1]. The mechanism underlying GPN development is complex. In 1977, Jannetta proposed that the short-circuit mechanism triggered by compression of the glossopharyngeal nerve due to blood vessels in the inlet region of the brainstem was the pathological basis of GPN [12]. In 1999, Matsushima inserted a microcatheter in the posterior inferior cerebellar artery area to induce GPN, confirming that vascular compression can cause GPN, which is consistent with the findings of a previous study [13]. Currently, there are several clinical treatments for GPN. The first approach involves the use of drugs, such as carbamazepine; however, it has limitations such as adverse effects, drug intolerance, and allergies. Another approach includes nerve blocking and surgery, which encompasses PRT, gamma knife surgery, rhizotomy (combining the glossopharyngeal nerve with the vagus nerve), and MVD [14].

Different nerve fibers have varying temperature tolerances, and in PRT, target nerve pain fibers are selectively destroyed by temperature adjustments, while touch and motion fibers are preserved. This mechanism not only reduces central nervous excitability but also maximizes the retention of nerve function, thereby improving patient satisfaction [15]. As one of the most commonly used techniques in pain diagnosis and treatment centers, PRT has been widely used for treating neuropathic pain, such as trigeminal neuralgia, GPN, and postherpetic neuralgia. Song et al. performed computed tomography (CT)-guided PRT in 117 patients with idiopathic GPN and found that 96 (82.1%) patients achieved “excellent” pain relief immediately following treatment, with a 5- and 10-year pain relief rate of 54.0% and 44.2%, respectively, indicating that PRT has immediate and long-term efficacy in treating GPN [16]. Although some patients in this study experienced recurrent pain symptoms after a few months, they still reported significant relief with therapeutic significance. Wang et al. performed CT-guided PRT in 71 patients with GPN and found that 63 (78.8%) patients experienced pain relief immediately after PRT. The proportion of patients who showed “excellent” or “good” pain relief at 1, 3, 5, and 10 years was 73.2%, 63.0%, 53.2%, and 43.0%, respectively [17]. The most common postoperative complications of PRT in treating GPN include sensory disturbance, difficulty swallowing, and gag reflex weakness; however, in the long-term follow-up of two large samples, these adverse reactions were substantially improved; these two studies confirmed that PRT has a direct and long-term curative effect on GPN treatment without any evident long-term complications [16, 17].

Previous studies [4] have shown that vessels in the cerebellopontine angle region compress the glossopharyngeal nerve root as it enters or exits the brainstem region, leading to the corresponding symptoms. This region, also known as the demyelinating area, serves as the transitional zone between central and peripheral myelin sheaths and lacks the protective wrapping of Schwann cells. The pulsatile stimulation can induce paroxysmal pain in the glossopharyngeal nerve distribution area, with the most common cause being the posterior inferior cerebellar artery [18, 19]. MVD should be performed under the guidance of a microscope to fully expose the glossopharyngeal and vagus nerve entry/exit points in the brainstem region for identifying responsible blood vessels. Teflon pads should be utilized to separate these blood vessels from the glossopharyngeal nerve, relieving vascular pressure on the nerve. To ensure an effective outcome, comprehensive decompression and exploration of the entire glossopharyngeal nerve should be undertaken, and any adherent arachnoid membrane should be gently relaxed during the decompression process [20]. MVD has been widely promoted and applied because of its definite curative effect and low complication rate. Xia et al. performed MVD in 228 patients with idiopathic GPN and found that 204 (89.5%) patients had an excellent outcome immediately after surgery; 107 patients were followed up for > 5 years, among whom 93 (86.9%) had excellent pain relief and 6 (5.6%) had good pain relief without any evident complications [21]. MVD is effective in treating GPN, but for some patients without evident responsible vessels, glossopharyngeal/vagal radiculotomy, with or without glossopharyngeal nerve MVD, serves as a safe and effective surgical treatment for GPN [22]. Moreover, a previous study compared the treatment plans of MVD only (22 cases) and MVD + glossopharyngeal radiculotomy (15 cases) in 37 patients with GPN and found no significant difference in the cure rate between the two groups; however, the incidence of complications was higher in the latter group than in the former group [20].

The present study demonstrated that both PRT and MVD provided immediate pain relief for patients with GPN. However, those who underwent MVD exhibited significantly lower pain scores starting from the first postoperative day. This difference may be attributed to the mechanism involving thermal coagulation through radiofrequency and microinjury resulting from localized surgery. MVD is a lengthy and complex procedure that necessitates a high level of expertise and must be performed under general anesthesia. Conversely, PRT serves as a suitable option for certain patients with GPN with underlying health conditions who cannot tolerate general anesthesia. We found that some patients who had undergone PRT experienced partial pain recurrence in the fourth week after surgery. Additionally, two young patients who had initially received PRT experienced more severe pain recurrence within just 1 year. However, one patient achieved significant pain relief with no recurrence after undergoing a second surgery with MVD. Considering the medium-term outcomes, MVD exhibited a higher complete relief rate than PRT, with no significant complications. It is recommended that for young patients with GPN, glossopharyngeal imaging should be performed to identify the vessels responsible for compression. If oral medication proves to be ineffective, MVD is suggested as it can provide a better and longer prognosis. MVD is also a reliable option for patients who experience short-term pain recurrence after PRT. Because PRT is conducted under local anesthesia, it has a shorter operative time, a briefer hospital stay, lower costs, and high efficacy. Therefore, it can be prioritized for elderly patients experiencing their first GPN episode or those with GPN without significant vascular compression evident in imaging. In terms of complications, some patients reported postoperative pharyngeal discomfort and weakened pharyngeal reflexes, all of which resolved over time without causing further harm. Hence, when choosing between the two surgical procedures, it is essential to consider the differences and select the optimal treatment plan based on the patients’ characteristics.

In summary, both PRT and MVD can relieve pain and improve sleep quality in patients with GPN. MVD has a higher complete cure rate but is costlier than PRT, and no significant difference in complications was observed between the two procedures. However, the main limitation of this study is that it only assessed the near- and mid-term efficacies of both procedures; moreover, this was a single-center study with a small sample size. Therefore, further multicenter studies with larger sample sizes should be conducted.

## Data Availability

The datasets of the current study are available from the corresponding authors upon reasonable request.
